# Supplementary material for: Origin of two-band chorus in the radiation belt of Earth
Source: Nat Commun. 2019 Oct 14;10:4672. doi: 10.1038/s41467-019-12561-3 (PMC6791895; doi:10.1038/s41467-019-12561-3)
Supplement: Supplementary file 1 — Supplementary Information [file 41467_2019_12561_MOESM1_ESM.docx]

Supplementary Information

**Origin of two-band chorus in the radiation belt of Earth**

Jinxing Li^1*^, Jacob Bortnik^1*^, Xin An^1^, Wen Li^2^, Vassilis Angelopoulos^3^, Richard M. Thorne^1^, Christopher T. Russell^3^, Binbin Ni^4,5^, Xiaochen Shen^2^, William S. Kurth^6^, George B. Hospodarsky^6^, David P. Hartley^6^, Herbert O. Funsten^7^, Harlan E. Spence^8^ and Daniel N. Baker^9^

^1^Department of Atmospheric and Oceanic Sciences, University of California, Los Angeles, California 90095, USA

^2^Center for Space Physics, Boston University, Boston, Massachusetts, USA.

^3^Department of Earth, Space and Planetary Sciences, University of California, Los Angeles, California 90095, USA

^4^Department of Space Physics, School of Electronic Information, Wuhan University, Wuhan 430072, Hubei, China

^5^CAS Center for Excellence in Comparative Planetology, Anhui, Hefei, China

^6^Department of Physics and Astronomy, University of Iowa, Iowa City, Iowa 52242-1479, USA

^7^ Los Alamos National Laboratory, MS-D466, PO Box 1663, Los Alamos, New Mexico 87545, USA

^8^ Institute for the Study of Earth, Oceans, and Space, University of New Hampshire, Durham, New Hampshire 03824-3525, USA

^9^Laboratory for Atmospheric and Space Physics, University of Colorado, Boulder, Colorado, USA.

Supplementary Discussion 1

**Electron distributions associated with banded chorus in low-density regions**

In low plasma density regions, the density cannot be reliably obtained because the upper hybrid frequency is below the HFR instrument threshold. Netherless, banded chorus are also observed to be associated with two anisotropic electron components. Supplementary Figure 3 shows a banded chorus event in which the upper-hybrid frequency cannot be clearly identified from the HFR measurements. The electrons PSD shows two anisotropic components: 0.3-5 keV and >30 keV, as well as a close-to-isotropic component at medium energies. An electron PSD plateau is seen at ~7 keV. In cases where banded chorus waves occur at very low density regions, the high-energy anisotropic electron components may exceed the upper energy limit of the HOPE instrument.

**Supplementary Discussion 2**

**Conjugate observations at low altitudes for the case study**

Chorus waves in Earth’s radiation belt can scatter tens of keV electrons to low altitudes. Supplementary Figure 4a shows the orbits of Van Allen Probe-A and two low-altitude (~800 km) satellites as a function of magnetic local time and L-shell during the occurrence of two-band chorus waves on 6 December 2015. Two polar orbiting probes, MetOp-01 and NOAA-19, belonging to the European Organization for the Exploitation of Meteorological Satellites (EUMETSAT) MetOp network and NOAA Polar Operational Environmental Satellites (POES), respectively, were roughly conjugate with Van Allen Probe-A. Each of them has two electron telescopes: a 0° telescope that measures electron flux within the loss cone, and a 90° telescope that measures the mirroring electron flux. Supplementary Figures 4b and 4e present electron fluxes at energies >30 keV (the lowest energy channel). The 0° telescope measurements by MetOp-01 and NOAA-19 indicate significant energetic electron precipitation, likely scattered by the two-band chorus waves observed at high altitudes.


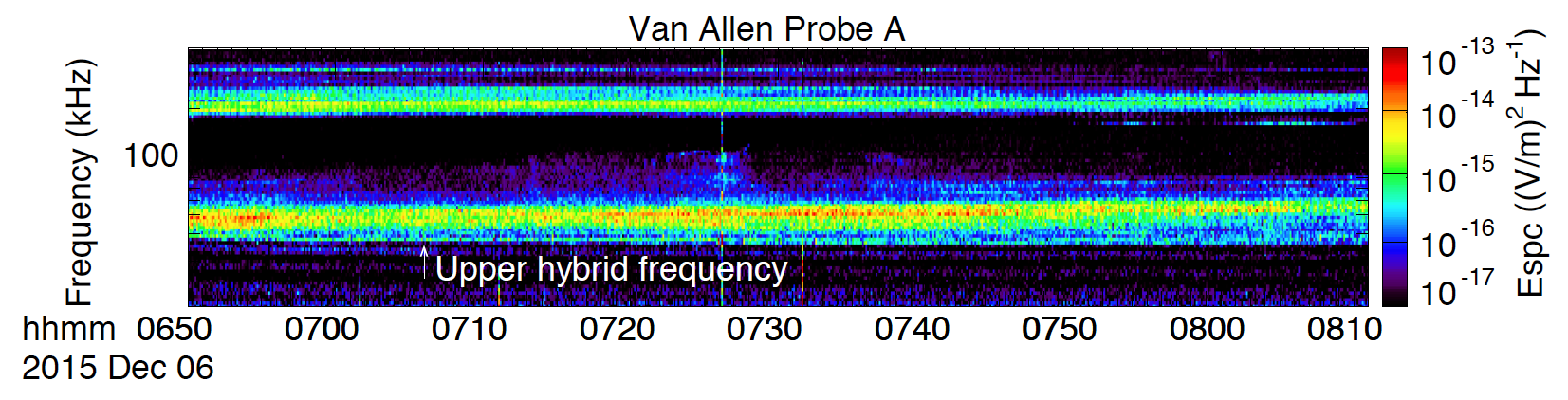


**Supplementary Figure 1.** **Van Allen Probe measurements of the upper hybrid frequency.**


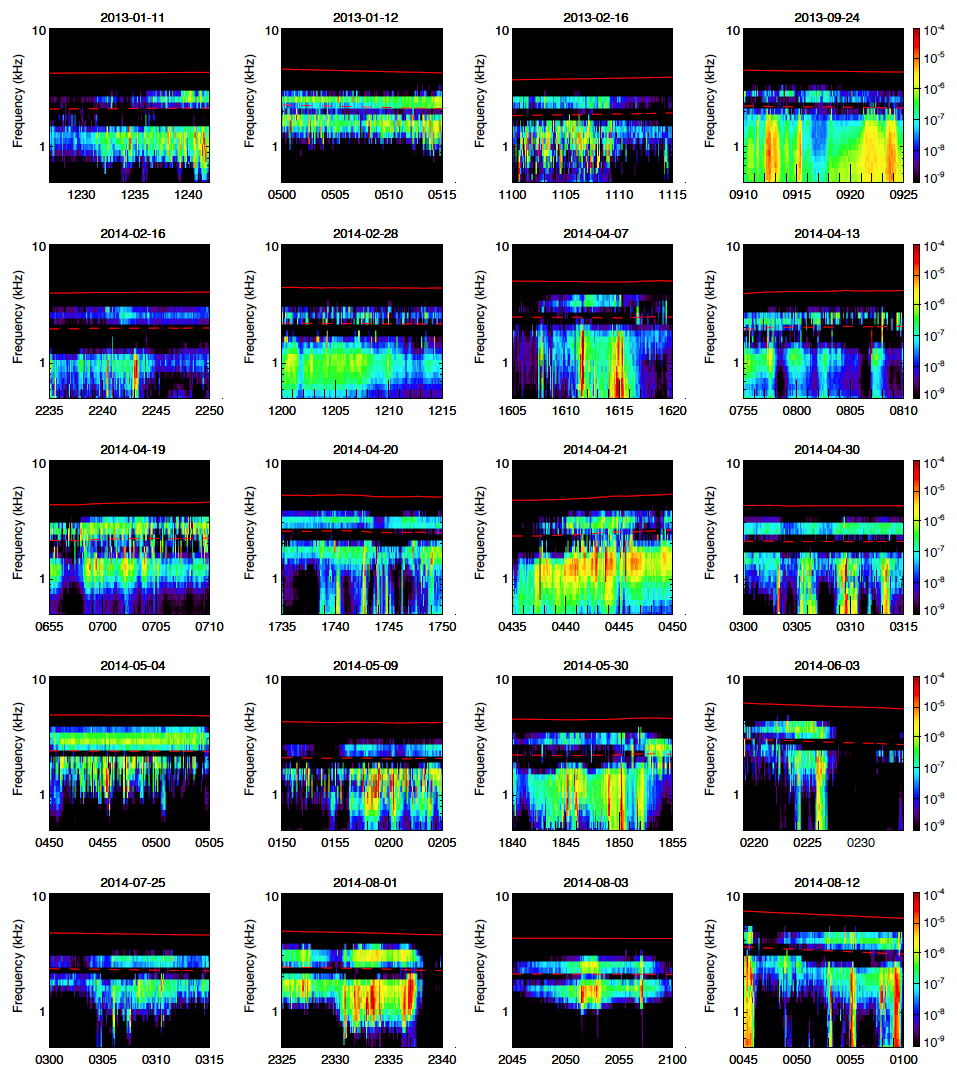
 Supplementary Figure 2. A list of two-band chorus events selected by our criteria. The selection criteria are: 1) the two-band chorus waves are observed longer than 5 minutes; 2) the plasma density (thus the Landau resonant energy) can be clearly determined from the upper hybrid frequency measurements; 3) observations in the same day are taken to be one event. The red solid and dashed lines represent *f*_ce_ and 0.5 *f*_ce_, respectively.


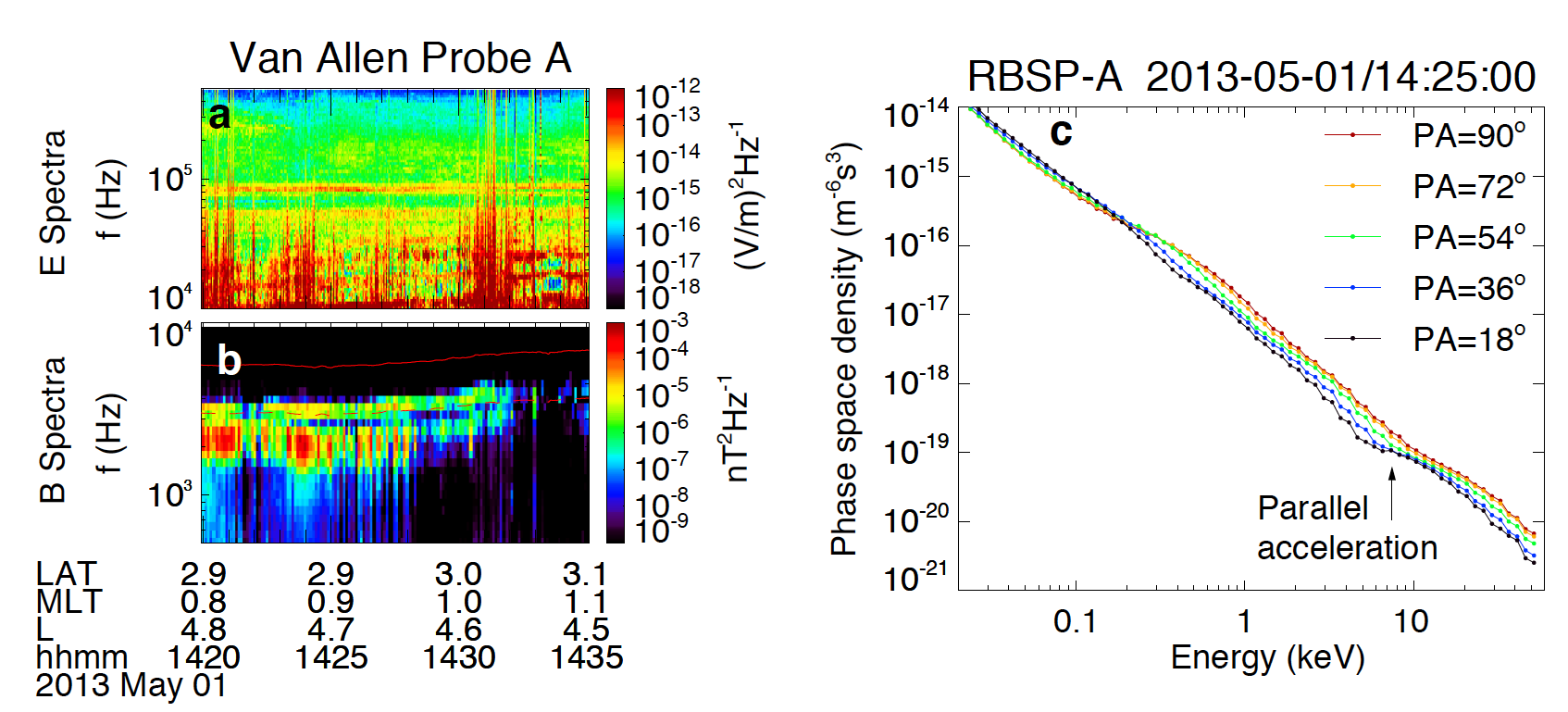


**Supplementary Figure 3.** **An example of banded chorus wave in the low-density region.** (a) The electric spectral intensity measured over 10-500 kHz. The upper-hybrid frequency cannot be clearly determined. (b) The magnetic spectral intensity measurement, showing banded chorus waves. (c) The electron PSD measurements, showing two anisotropic components and a PSD plateau at ~7 keV.


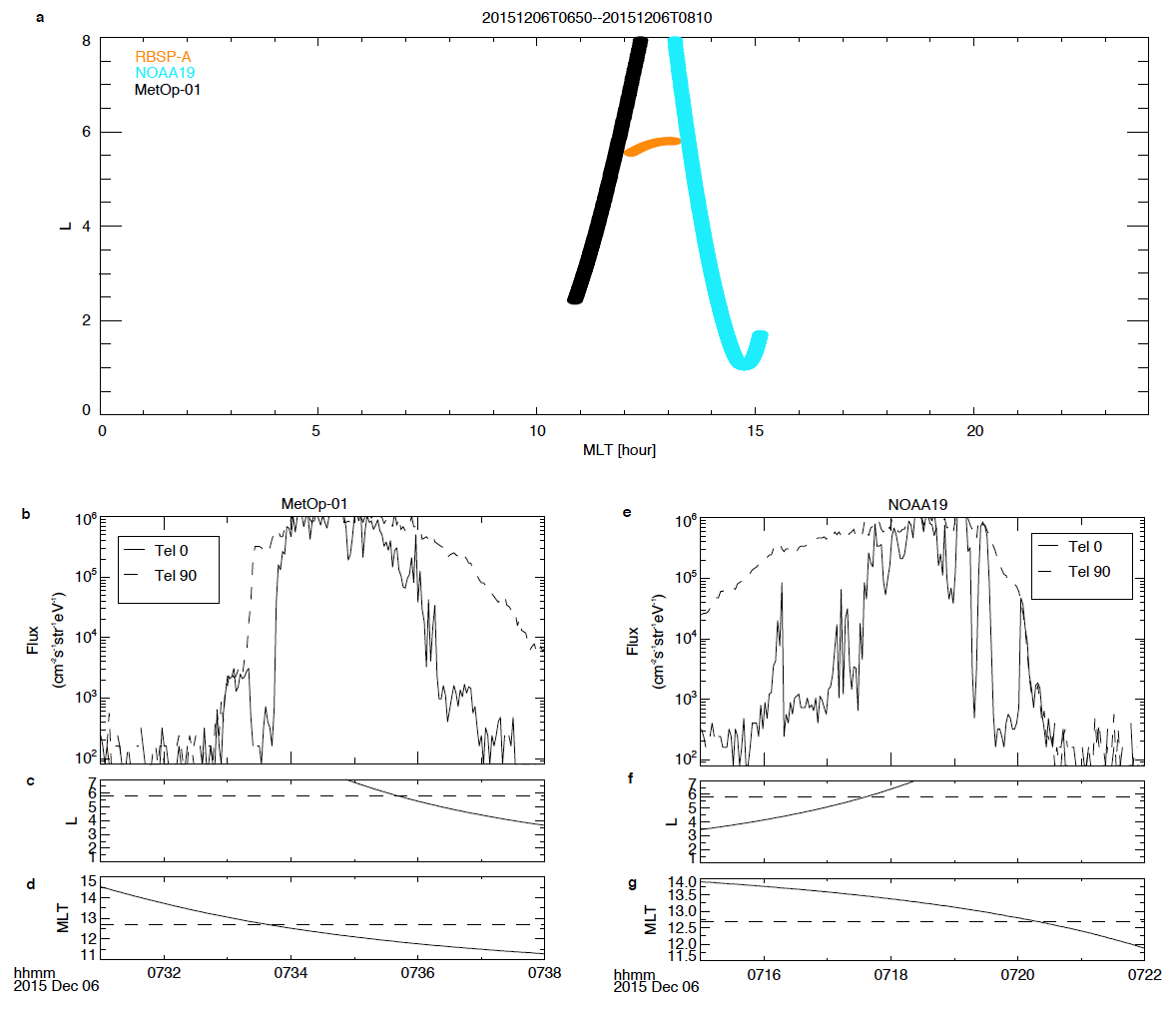


Supplementary Figure 4. Electron precipitation measured by MetOp-01 and NOAA-19 spacecraft. (A) The spacecraft orbit mapped to magnetic local time and L-shell from 06:50 to 08:10 UT on 6 December 2015, showing good conjugation between Van Allen Probe-A and two polar orbiting probes, NOAA-19 and MetOp-01. (B) Flux of >30 keV electrons measured by two telescopes onboard MetOp-01: Tel 0, which measures electrons within the loss cone, and Tel 90, which measures trapped electrons. (C) The L-shell value and (D) the magnetic local time of the spacecraft. (E-G) The same as in Figures B-D but the data is from the NOAA19 spacecraft. Dashed lines in panels C and F (D and G) mark the average L-shell (MLT) of Van Allen Probe-A.


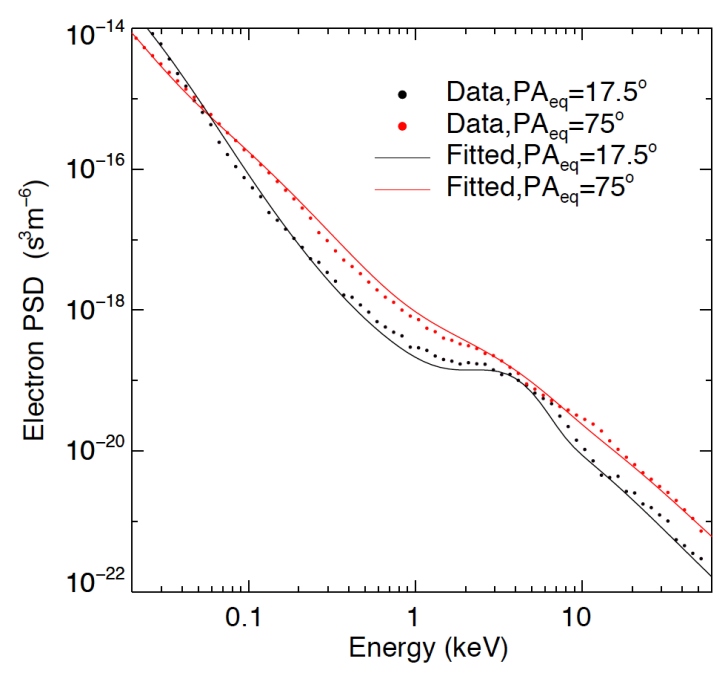


**Supplementary Figure 5.** **A comparison between the measured and fitted electron PSD**. We note that in the present study, the averaged spacecraft magnetic latitude during 07:00-08:00 UT was -6.2°. Thus, the local pitch angles of 18° and 90° correspond to equatorial pitch angles of 17.5° and 77°, respectively.


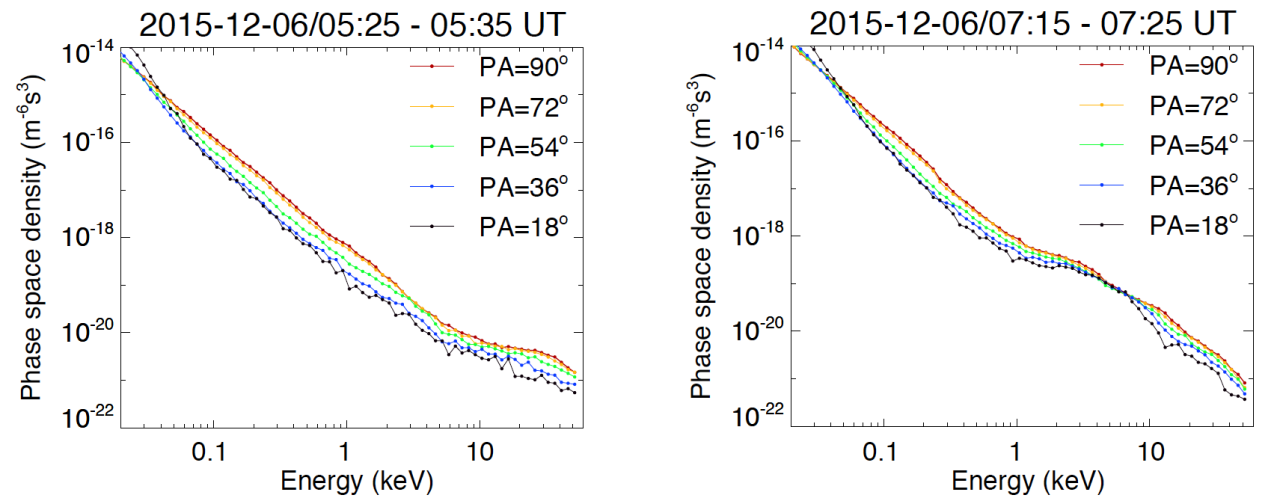


**Supplementary Figure 6. Electron PSD associated with one-band and two-band chorus.** The wave spectral intensities of these two events are shown in Figure 5. The electron PSD distributions associated with one-band chorus waves exhibit one anisotropic component, while those associated with two-band chorus exhibit two anisotropic components.
