# Supplementary material for: Origin of two-band chorus in the radiation belt of Earth
Source: Nat Commun. 2019 Oct 14;10:4672. doi: 10.1038/s41467-019-12561-3 (PMC6791895; doi:10.1038/s41467-019-12561-3)
Supplement: Supplementary file 2 — Description of Additional Supplementary Files [file 41467_2019_12561_MOESM2_ESM.pdf]

Title: Supplementary Movie 1

Description: Naturally occurring chorus radio waves in space consist of two bands in frequency, and each band consists of rising-tone emissions. They are widely publicized as “Earthsong”. This video shows a dynamic spectrogram of chorus waves recorded by NASA’s Van Allen Probe, with a cursor indicating the time position of the audio track.
